# Plasma Proteome Biomarkers of Inflammation in School Aged Children in Nepal

**DOI:** 10.1371/journal.pone.0144279

**Published:** 2015-12-04

**Authors:** Sun Eun Lee, Keith P. West, Robert N. Cole, Kerry J. Schulze, Parul Christian, Lee Shu-Fune Wu, James D. Yager, John Groopman, Ingo Ruczinski

**Affiliations:** 1 Center for Human Nutrition, Department of International Health, Johns Hopkins Bloomberg School of Public Health, Baltimore, Maryland, United States of America; 2 Mass Spectrometry and Proteomics Facility, Department of Biological Chemistry, Johns Hopkins School of Medicine, Baltimore, Maryland, United States of America; 3 Department of Environmental Health Sciences, Johns Hopkins Bloomberg School of Public Health, Baltimore, Maryland, United States of America; 4 Department of Biostatistics, Johns Hopkins Bloomberg School of Public Health, Baltimore, Maryland, United States of America; University of California, San Diego, UNITED STATES

## Abstract

Inflammation is a condition stemming from complex host defense and tissue repair mechanisms, often simply characterized by plasma levels of a single acute reactant. We attempted to identify candidate biomarkers of systemic inflammation within the plasma proteome. We applied quantitative proteomics using isobaric mass tags (iTRAQ) tandem mass spectrometry to quantify proteins in plasma of 500 Nepalese children 6–8 years of age. We evaluated those that co-vary with inflammation, indexed by α-1-acid glycoprotein (AGP), a conventional biomarker of inflammation in population studies. Among 982 proteins quantified in >10% of samples, 99 were strongly associated with AGP at a family-wise error rate of 0.1%. Magnitude and significance of association varied more among proteins positively (n = 41) than negatively associated (n = 58) with AGP. The former included known positive acute phase proteins including C-reactive protein, serum amyloid A, complement components, protease inhibitors, transport proteins with anti-oxidative activity, and numerous unexpected intracellular signaling molecules. Negatively associated proteins exhibited distinct differences in abundance between secretory hepatic proteins involved in transporting or binding lipids, micronutrients (vitamin A and calcium), growth factors and sex hormones, and proteins of largely extra-hepatic origin involved in the formation and metabolic regulation of extracellular matrix. With the same analytical approach and the significance threshold, seventy-two out of the 99 proteins were commonly associated with CRP, an established biomarker of inflammation, suggesting the validity of the identified proteins. Our findings have revealed a vast plasma proteome within a free-living population of children that comprise functional biomarkers of homeostatic and induced host defense, nutrient metabolism and tissue repair, representing a set of plasma proteins that may be used to assess dynamics and extent of inflammation for future clinical and public health application.

## Introduction

Inflammation is an evolutionarily conserved body response for protecting the host from potentially lethal stresses [1]. It is often understood as a process to isolate and eliminate pathogens or other non-self or intolerant agents by immune cells [2] followed by processes that resolve inflammation, repair damaged tissues and restore homeostasis [3]. However, when the transition to tissue repair fails, inflammatory processes can persist and cause harm [4]. It is well documented that chronic inflammation, often viewed as subclinical, contributes to chronic disease processes leading to obesity, diabetes, atherosclerosis, rheumatoid arthritis and cancer [4]. In addition, inflammation in response to continuous exposure to infectious agents [5–7] and environmental toxins [8, 9] may contribute to childhood undernutrition and developmental deficits in impoverished areas of the world where poor sanitation and frequent infections are common.

An observable characteristic of inflammation is an increase or decrease in the release and concentration of certain proteins in the bloodstream, a process regarded as an acute phase reaction, although it can occur in response to either acute or chronic inflammatory stress [1]. At least 50 acute phase proteins (APP) are known to increase or decrease by at least 25% in humans, a commonly used cut-off, from their baseline concentrations during inflammation [10, 11]. This quantitative alteration is mainly regulated by inflammatory mediators that induce reactions within innate and adaptive immune, neuroendocrine, vascular endothelial, hematopoietic, metabolic, and other defense and repair systems [10]. Due to these systemic effects, APPs are widely used as clinical diagnostic and prognostic indicators of disease processes [12]. Also, APPs are often recommended for use in population studies as correction factors for assessing status with respect to micronutrients, whose indicator concentrations may decrease (e.g., serum retinol for vitamin A) or rise (e.g, serum ferritin for iron) with inflammation [13–15]. Although large in number, only a few APP, primarily α-1-acid glycoprotein (AGP) and C-reactive protein (CRP), are typically assessed in population studies as indicators of inflammation [15]. This is because their responses and prognostic values are different and well-defined, with concentrations that reliably increase during infection, responding to numerous systemic infections and other pathological conditions, and decrease with resolution [10, 14, 16]. Yet this nearly sole reliance on AGP and CRP may also, in part, be due to an incomplete understanding of the many and varied molecular mediators and responders to inflammation, a general inability to measure and interpret their responses to inflammatory stresses, limited resources to quantify multiple APPs, and a general lack of awareness in the public health research community of the highly varied dynamics of inflammation that coexist in populations.

The unbiased approach of plasma proteomics offers a unique opportunity to discover, quantify and explore the utility of a wider array of proteins that respond to, initiate, maintain and resolve inflammation. In a given population, all phases of the inflammatory response are likely to be present, highlighting the potential utility of accessing and interpreting a larger inventory of biomarkers with which to understand homeostatic mechanisms, disease processes, types of inflammation and the range of inflammatory exposures in an environment.

In this population study of 6–8 year old Nepalese children [17], we rely primarily on the circulating concentration of AGP to define inflammation as a continuous measure. AGP, or orosomucoid (ORM), is present in plasma as a mixture of ORM1 and ORM2 each of which is encoded by two tandomly arranged genes, *ORM1* and *ORM2* [18]. Because the concentration of AGP slowly rises and remains elevated during recovery or convalescence, AGP may be considered more sensitive in detecting chronic and subclinical inflammation in populations than CRP, which tends to react, spike and resolve more quickly, and thus be less often detected in prevalence surveys [14]. We refer to the entire set of plasma proteins that definitively and quantitatively co-vary with AGP, as a “population plasma inflammasome” whose members may include but extend beyond classical acute phase proteins, contain proteins of hepatic origin as well as those secreted or leaked from extra-hepatic tissues [19, 20], and include proteins involved in constitutive and induced homeostatic immune, coagulative and repair processes associated with inflammation. We verify the reliability of this set of inflammatory proteins by repeating the same analysis using CRP, and evaluating overlap. We distinguish the compound term, population plasma inflammasome, from “inflammasome”, used to describe an intracellular multiprotein complex that is responsible for activating the processing of pro-inflammatory cytokines [21]. While limited animal and human subject experimentations have described an endotoxin-induced plasma inflammasome and compilations of inflammatory plasma proteins from databases for clinical use exist in the literature [22–24], definition of a human population plasma inflammasome remains incomplete.

In this population sample [17], 31% and 6% of children exhibited elevated plasma concentrations of AGP and CRP above conventional, clinical disease thresholds of 1g/L and 5 mg/L, respectively, reflecting a population affected by chronic inflammation [13] but not acutely ill, providing an opportunity to explore markers of low-grade (“subclinical”) inflammation that may nonetheless be useful for population assessment [25]. We hypothesized the existence of a quantifiable population plasma inflammasome, defined as a suite of plasma proteins tracking AGP with high confidence, which we verified by also evaluating their concordance of association with plasma CRP concentrations.

## Materials and Methods

### Field study and assessment of inflammatory biomarkers

In 1999–2001, a community-randomized, placebo-controlled field trial was carried out in the District of Sarlahi, Nepal to assess effects of 4 combinations of antenatal supplemental micronutrients on birth outcomes [26]. Of 3,892 children exiting the trial as infants, 3,524 were followed-up at 6–8 years of age for a nutritional, health and socio-demographic assessment by methods previously described [27, 28].

During home visits, children of consenting parents were asked to fast overnight, and phlebotomists collected venous blood samples the next morning from 94% (n = 3,305) of eligible children. Bio-specimens were brought to a field laboratory for plasma extraction. Plasma samples were stored in liquid nitrogen tanks and shipped to the Center for Human Nutrition, Johns Hopkins Bloomberg School of Public Health in Baltimore, MD, USA. Among the plasma samples, 2,130 samples (64%) were selected based on having multiple plasma aliquots, complete epidemiologic data from both the original trial and follow-up assessment, and valid birth size measures (birth weight measured <72 hours after birth). Of the 2,130, 1,000 child plasma samples were randomly sampled across the five original maternal intervention groups (n = 200 from each) and analyzed for multiple micronutrients and inflammation (AGP and CRP) status by conventional assays [13]. Specifically, concentrations of plasma AGP and CRP were measured by a radial immunodiffusion assay (Kent Laboratories; CV = 10.0%) and a benchtop clinical chemistry analyzer (Immulite 1000; Siemens Diagnostics; CV = 5.8%), respectively. From the 1,000 specimens, specimens were ordered by date of field blood collection within each original maternal supplement allocation stratum of 200 specimens, and following a chance start every other specimen was selected for inclusion into the proteomics archive, yielding a total of 500 samples (n = 100 per maternal group) [17]. Socio-demographic, anthropometric and morbidity status, and dietary intakes among the 500 selected children were comparable to the 500 not in the proteomics study [13].

### Ethics statement

The original prenatal micronutrient supplementation trial was approved by the institutional review board (IRB) at Johns Hopkins University, Baltimore, MD, USA and the Nepal Health Research Council (NHRC) in Kathmandu, Nepal and registered with ClinicalTrials.gov: NCT00115271. The follow-up study with biospecimen collection was approved by the IRB and NHRC. During follow-up home visits, oral consent was obtained from mothers of eligible children after being explained the purpose, activities and risks of participation by trained field staff following an approved script. Oral consent was deemed appropriate due to high levels of illiteracy in the study population. Consent was documented by field staff and entered into the study database.

### Proteomics and data analysis

Immuno-depletion and proteomics assays have been previously described [17]. Briefly, a master plasma pool was prepared by combining plasma aliquots of 25 μL from each of the 1,000 samples comprising the micronutrient archive. The 500 specimens selected for proteomics analysis each comprised 40 μL of plasma. Individual specimens plus master pool aliquots were depleted of six high abundance proteins (albumin, transferrin, IgG, IgA, anti-trypsin, and haptoglobin) using a Human-6 Multiple Affinity Removal System LC column (Agilent Technologies). Proteomics analysis was performed at the Proteomics and Mass Spectrometry Core within the Johns Hopkins School of Medicine. Immno-depleted samples (100 μg of protein) were digested overnight with trypsin (Promega, sequencing grade). Peptide samples of 7 individuals and one masterpool sample were randomly labeled with 8-plex Isobaric Tag for Relative and Absolute Quantification (iTRAQ) reagents that contained different reporter ions which can be used as measures of peptide relative abundance in the original sample. The combined sample was fractionated into 24 fractions by strong cation exchange chromatography. iTRAQ-labeled peptides were loaded on to a reverse-phase nanobore column. Eluted peptides were sprayed into an LTQ Orbitrap Velos mass spectrometer (Thermo Scientific) and interfaced with a NanoAcquity ultra-HPLC (Waters). Full MS scans and fragmented MS/MS scans were acquired and these spectra were searched against Refseq 40 protein database using MASCOT (Matrix Science v2.3) through Proteome Discoverer software (v1.3, Thermo Scientific). Peptides were identified with a confidence threshold of <5% false discovery rate. A total of 72 iTRAQ experiments were performed for this study.

Details of relative abundance estimation have been described previously [29]. Briefly, reporter ion intensities were log_2_ base-transformed and median normalized for each reporter ion intensity spectrum. The relative abundance of proteins in each channel of each experiment was estimated by calculating the median of all the median-polished log_2_ ion intensities across all spectra belonging to each protein. Corrections for differences in amounts of material loaded in the channels and sample processing were carried out by subtracting the channel median from the relative abundance estimate, normalizing all channels to have median zero. Because there is no physiologically meaningful cut-off of plasma AGP concentration to dichotomize inflammation status, we employed linear mixed-effects (LME) models to assess the linear association between log_2_ transformed plasma AGP concentration and relative abundance of individual plasma proteins from multiple iTRAQ experiments. A univariate random intercept model was fit for each protein with AGP as a dependent variable, the protein as a fixed effect, and each iTRAQ experiment as a random effect [28]. Model parameters in these mixed effects models were estimated via Restricted Maximum Likelihood [30]. Estimates of absolute protein abundance were calculated as Best Linear Unbiased Predictors [31].

Observed sample sizes were different among proteins due to missing values from the mass-spectrometry [32]. We report summary statistics for the association between plasma protein abundance and AGP as percent change in AGP per 2-fold (100%) increase in protein relative abundance (derived from the slope of the LME model) and its statistical significance (p-value), and the correlation between estimated absolute protein abundance and plasma AGP concentration. We controlled the family-wise error rate (FWER) at the 0.1% level using a Bonferroni correction, to only select proteins truly associated with AGP (*P* <1.02e-06). We also present in Supporting Information, proteins passing a false discovery rate threshold of 1% (q<0.01).

Additional analyses were conducted to examine the validity of the identified proteins. Specifically, we applied the same analytical approach and significance threshold to identify, quantify and evaluate the direction and strength of association between plasma protein relative abundance and CRP concentration. In addition, we identified differentially abundant proteins between children who reported at least one day of symptomatic morbidity (fever, diarrhea, productive cough, or rapid breathing) and children without any symptoms in the week prior to blood sampling. All CRP and morbidity-related results are presented in Supporting Information tables.

Since we expected that proteins associated with AGP might be co-regulated or co-vary in the same biological systems, we examined relationships between proteins by constructing a correlation matrix and performed principal component analysis (PCA). Pairwise protein:protein correlation coefficients were calculated within each iTRAQ experiment and averaged coefficients across iTRAQ experiments to construct a correlation matrix and to perform PCA. Bi-plots were constructed to visualize the 1^st^, 2^nd^, and 3^rd^ principal components of each protein from PCA [33].

Corresponding gene symbols of protein genInfo identifier (gi) numbers were derived from the Human Genome Organisation (HUGO) gene annotation and used in tables and figures, once linked to protein names in initial descriptive tables, to conserve space [34]. Resources of general description of proteins including cellular compartment, biological/molecular functions, and mRNA expression across tissues were extracted from the NCBI protein database, UniProt beta, Gene ontology Annotation (Uniprot-GOA) database, BioGPS, COMPARTMENTS, and in-depth review of literature [35–39].

All analyses were performed using the R Environment for Statistical Computing (version 3.1.0; R Foundation for Statistical Computing, Vienna, Austria).

## Results

### Study participant characteristics

Demographic, nutritional, and health characteristics of study children (Table 1) were similar to children in the original, larger follow-up cohort [28]. Children were undernourished, compared to the WHO reference population, reflected by prevalence rates of 40%, 15%, and 50% for stunting, thinness, and underweight. More than half and approximately 30% of children consumed dairy food and dark green leafy vegetables equal to or greater than 3 times in the past week, respectively, but meat, fish, and eggs were less frequently consumed by children. Approximately 8% of children reported at least one episode of fever in the past week, but the prevalence of other symptoms was low (<5%). Fourteen percent of children reported any symptoms of fever, diarrhea, productive cough, or rapid breathing in the past week. Median (interquartile range) of plasma AGP and CRP concentrations were 0.84 (0.70, 1.05) g/L and 0.28 (0.13, 0.78), with 30% and 6% of children having an elevated AGP concentration (>1 g/L) and CRP (>5 mg/L) concentrations, respectively.

**Table 1:** Demographic, nutritional, and health characteristics of 6–8 year old children in rural Nepal (n = 500).^a^

Characteristics	Values
Age, years	7.5 (0.4)
Girls, %	50.2
Ethnicity (Pahadi), %	31.8
**Anthropometric measurements** ^b^	
Height, cm	114.1 (5.8)
Weight, kg	18.2 (2.2)
BMI, kg/m^2^	14.0 (1.0)
Mid-upper arm circumference, cm	15.4 (1.1)
Height-for-age	-1.77 (0.95)
BMI-for-age	-1.20 (0.89)
Weight-for-age	-1.99 (0.85)
Stunted, %^c^	39.1
Thin, %^c^	16.4
Underweight, %^c^	48.5
**Dietary, ≥3 intake in the past week, %**	
Dairy	56.6
Meat	8.6
Fish	9.0
Eggs	2.2
Dark green leafy vegetables	32.4
**Morbidity, symptoms reported in the past week, %**	
Fever	8.2
Diarrhea	3.2
Productive cough	3.8
Rapid breathing	2.8
Any of above symptoms	14.0
**Plasma Concentration of AGP**	
AGP, g/L	0.84 (0.70, 1.05)^d^
AGP > 1.0 g/L^e^, %	29.8
CRP, mg/L	0.28 (0.13, 0.78)^d^
CRP > 5.0 mg/L^e^, %	6.0

Abbreviations: AGP, α-1-acid glycoprotein; BMI, body mass index; CRP, C-reactive protein.

^a^Values are means (SD) or percentages (95% CI).

^b^One outlier was excluded (n = 499).

^c^Z-scores were calculated based on World Health Organization reference for 5–19 years. Underweight, weight-for-age Z-score< -2; stunted, height-for-age Z-score< -2; thin, BMI-for-age Z-score< -2 [40].

^d^Median (Interquartile range).

^e^Cut-off for inflammation [41].

### Plasma proteins associated with AGP

**Fig 1:**
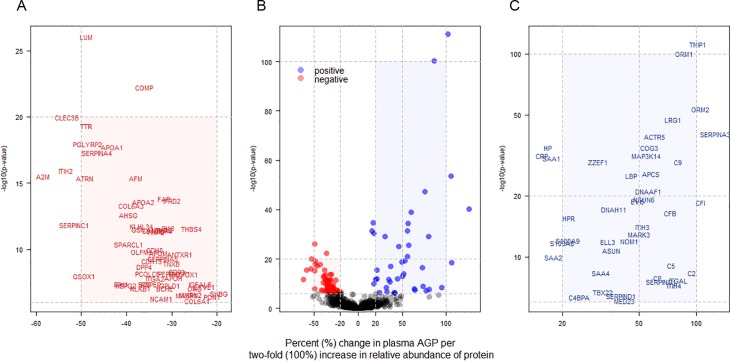
Volcano plot of plasma proteins associated with plasma α-1-acid glycoprotein (AGP) in 6–8 year old children in rural Nepal. Plot (A) and (C) are enlarged rectangles in plot (B). (A) Plasma proteins negatively associated with AGP, presented by gene symbol (n = 58); (B) Plasma proteins associated with AGP were colored in red and blue (n = 99); (C) Plasma proteins positively associated with AGP, presented by gene symbol (n = 41). x- and y-axes are logarithmic.

A total of 3,933 proteins were identified and quantified among 72 iTRAQ experiments required to analyze the 500 child plasma samples, of which 982 proteins were quantified in >10% of all samples (n>50). Ninety-nine proteins (~10% of all proteins adequately quantified) significantly co-varied with plasma AGP passing a Bonferroni corrected significance level, of which 41 and 58 proteins were positively and negatively associated with AGP, respectively. Among proteins positively associated with AGP, TNFAIP3 interacting protein 1 (Gene symbol: TNIP1) (*P* = 7.6x10^-112^) and orosomucoid 1 (ORM1) (*P* = 4.6x10^-101^) showed the strongest associations, followed by orosomucoid 2 (ORM2) (*P* = 3.1x10^-54^) (Table 2). Lumican (LUM) (*P* = 9.1x10^-27^) and cartilage oligomeric matrix protein (COMP) (*P* = 5.7x10^-23^) were most strongly associated among negative correlates (Table 3). A volcano plot shows distinct patterns within the population plasma inflammasome (Fig 1A–1C). Specifically, the percent change in AGP (95% confidence intervals) and strength of significance of association varied more widely within the group of positively than negatively associated proteins. A 86.6 (76.2, 97.6)% and 105.3 (87.4, 124.8)% increase in AGP concentration was associated with a 100% (two-fold) increase in relative abundance of ORM1 and ORM2, respectively (Fig 1C). A comparable 73~105% increase in AGP was also associated with a 100% increase in complement components 2, 5, and 9 (C2/5/9) and complement factors F and I (CFB and CFI). On the other hand, a smaller 15~18% increase in AGP was associated with a 2-fold rise in other acute phase proteins such as CRP, haptoglobin (HP), and serum amyloid A 1 and 2 (SAA1/2) (Fig 1C). Overall, a narrower range in reduction in AGP, 20~40%, was associated with a 2-fold increase in the relative abundance of 46 out of the 58 negatively associated proteins (Fig 1A). A total of 206 plasma proteins passed a false discovery significance threshold of q<0.01 (~20% of all analyzed), representing a larger plasma proteome that appears to covary with plasma AGP, as listed in Supporting Information (S1 and S2 Tables).

**Table 2:** Plasma proteins positively associated with plasma α-1-acid glycoprotein (AGP) in 6–8 year old children in rural Nepal, ordered by *P*.^a^

Protein name	Gene symbol	n^b^	% change in AGP (95% CI)^c^	*P* ^d^	q^e^	r^f^	Accession^g^
TNFAIP3-interacting protein 1	TNIP1	388	101.8 (89.8, 114.6)	7.6E-112	8.4E-109	0.82	116256481
Orosomucoid 1	ORM1	500	86.6 (76.2, 97.6)	4.6E-101	2.5E-98	0.77	167857790
Orosomucoid 2	ORM2	500	105.3 (87.4, 124.8)	3.1E-54	1.1E-51	0.67	4505529
Leucine-rich alpha-2-glycoprotein 1	LRG1	500	75.5 (62.7, 89.4)	5.6E-48	1.5E-45	0.65	16418467
Serpin peptidase inhibitor, clade A, Member 3	SERPINA3	500	125.9 (100.4, 154.5)	7.0E-41	1.5E-38	0.63	50659080
Actin-related protein 5	ACTR5	255	60.4 (49.5, 72.2)	9.6E-40	1.8E-37	0.74	151301041
Haptoglobin isoform 1	HP	354	16.8 (14.0, 19.7)	2.7E-35	4.0E-33	0.66	4826762
Conserved oligomeric golgi complex subunit 3	COG3	215	56.8 (46.0, 68.4)	2.9E-35	4.0E-33	0.73	13899251
C-reactive protein, pentraxin-related	CRP	438	15.6 (12.8, 18.4)	3.2E-32	3.5E-30	0.61	55770842
Mitogen-activated protein kinase kinase kinase 14	MAP3K14	307	55.6 (44.5, 67.5)	3.2E-32	3.5E-30	0.66	115298645
Serum amyloid A protein	SAA1	493	17.6 (14.4, 20.9)	3.1E-31	3.1E-29	0.59	40316912
Complement component 9	C9	500	80.2 (62.8, 99.6)	6.0E-30	5.3E-28	0.58	4502511
Zinc finger ZZ-type and EF-hand domain-containing protein 1	ZZEF1	444	30.7 (24.8, 36.9)	6.2E-30	5.3E-28	0.60	73747881
Serum amyloid P component	APCS	500	57.2 (44.5, 70.9)	3.0E-26	2.2E-24	0.56	4502133
Lipopolysaccharide-binding protein	LBP	500	45.7 (35.8, 56.4)	9.9E-26	6.7E-24	0.56	31652249
Leucine-rich repeat-containing protein 50	DNAAF1	207	56.2 (42.5, 71.2)	9.4E-22	5.1E-20	0.69	157674358
Putative methyltransferase NSUN6	NSUN6	249	53.4 (39.9, 68.2)	6.4E-20	3.1E-18	0.60	32698918
Ecotropic viral integration site 5 protein homolog	EVI5	271	49.5 (37.0, 63.2)	1.6E-19	7.1E-18	0.55	68299759
Complement factor I	CFI	500	105.7 (75.6, 140.9)	3.4E-19	1.5E-17	0.52	119392081
Dynein heavy chain 11, axonemal	DNAH11	298	37.1 (27.5, 47.4)	8.4E-18	3.2E-16	0.58	51479173
Complement factor B	CFB	500	72.9 (52.2, 96.4)	3.6E-17	1.3E-15	0.51	67782358
Haptoglobin-related protein	HPR	431	21.6 (16.0, 27.4)	3.0E-16	1.0E-14	0.51	45580723
Inter-alpha (globulin) inhibitor H3	ITIH3	500	51.8 (36.6, 68.7)	8.0E-15	2.4E-13	0.49	133925809
MAP/Microtubule affinity-regulating kinase 3 isoform E	MARK3	231	50.3 (34.9, 67.5)	1.1E-13	3.0E-12	0.56	193083131
Protein S100-A9	S100A9	500	21.3 (15.1, 28.0)	8.9E-13	2.2E-11	0.48	4506773
Nucleolar MIF4G domain-containing protein 1	NOM1	119	44.8 (30.6, 60.5)	1.1E-12	2.7E-11	0.62	61097912
RNA polymerase II elongation factor ELL3	ELL3	214	34.5 (23.8, 46.0)	1.4E-12	3.4E-11	0.54	13376768
Protein S100-A8	S100A8	500	20.0 (14.0, 26.3)	2.2E-12	4.9E-11	0.47	21614544
Cell cycle regulator mat89Bb homolog	ASUN	235	36.2 (24.4, 49.1)	1.9E-11	3.6E-10	0.52	155030185
Serum amyloid A2 isoform a	SAA2	189	18.0 (12.2, 24.1)	1.0E-10	1.9E-09	0.55	188497671
Complement component 5	C5	500	73.8 (45.6, 107.5)	8.7E-10	1.4E-08	0.45	38016947
Complement component 2 isoform 1	C2	423	94.8 (55.8, 143.6)	4.5E-09	7.0E-08	0.45	14550407
Serum amyloid A-4 protein	SAA4	493	31.8 (20.1, 44.5)	4.6E-09	7.1E-08	0.44	10835095
Ceruloplasmin	CP	500	63.8 (38.2, 94.3)	1.3E-08	1.8E-07	0.44	4557485
Integrin alpha L isoform B	ITGAL	82	80.5 (46.3, 122.7)	2.3E-08	3.0E-07	0.65	167466217
Serpin peptidase inhibitor, clade G, member 1	SERPING1	500	64.7 (38.0, 96.5)	2.9E-08	3.8E-07	0.44	73858570
Inter-alpha (globulin) inhibitor H4 isoform 1	ITIH4	500	76.9 (43.9, 117.5)	5.6E-08	6.9E-07	0.44	31542984
T-box transcription factor TBX22 isoform 1	TBX22	103	32.5 (19.0, 47.5)	2.0E-07	2.4E-06	0.46	18375603
Heparin cofactor II	SERPIND1	500	40.9 (23.4, 60.9)	3.6E-07	4.1E-06	0.43	73858566
Complement component 4 binding protein, alpha chain	C4BPA	500	24.4 (14.2, 35.4)	4.6E-07	5.1E-06	0.43	4502503
Mediator of RNA polymerase II transcription subunit 23 isoform b	MED23	196	41.7 (23.2, 62.9)	8.6E-07	9.2E-06	0.48	28558969

^a^Plasma proteins that achieved a Bonferroni corrected significance level (*P* <0.001/982 = 1.02e-06).

^b^The number of child plasma samples of each listed protein (50<n≤500).

^c^Percent change (%) in plasma AGP per two-fold (100%) increase in relative abundance of protein.

^d^ *P* value for the hypothesis test of null association between plasma AGP and protein.

^e^Adjusted *P* value correcting multiple hypothesis testing (false discovery rate).

^f^Correlation between plasma AGP concentration and protein.

^g^GenInfo Identifier for protein sequence assigned by the National Center for Biotechnology Information.

**Table 3:** Plasma proteins negatively associated with plasma alpha-1-acid glycoprotein (AGP) in 6–8 year old children in rural Nepal, ordered by *P*.^a^

Protein name	Gene symbol	n^b^	% change in AGP (95% CI)^c^	*P* ^d^	q^e^	r^f^	Accession^g^
Lumican	LUM	500	-48.9 (-54.8, -42.2)	9.1E-27	7.1E-25	-0.56	4505047
Cartilage oligomeric matrix protein	COMP	500	-36 (-41.4, -30.0)	5.7E-23	3.5E-21	-0.54	40217843
Tetranectin	CLEC3B	500	-53 (-60.0, -44.9)	1.0E-20	5.4E-19	-0.53	156627579
Transthyretin	TTR	500	-48.8 (-55.7, -40.9)	4.7E-20	2.3E-18	-0.52	4507725
N-acetylmuramoyl-l-alanine amidase	PGLYRP2	500	-48.6 (-55.7, -40.4)	1.2E-18	4.9E-17	-0.52	156616294
Apolipoprotein A-I	APOA1	500	-43.2 (-49.9, -35.5)	1.9E-18	7.6E-17	-0.52	4557321
Serine proteinase inhibitor, clade a (alpha-1 antiproteinase, antitrypsin), member 4	SERPINA4	500	-46.5 (-53.6, -38.4)	4.9E-18	1.9E-16	-0.51	21361302
Inter-alpha globulin inhibitor h2 polypeptide	ITIH2	500	-53.4 (-61.1, -44.2)	1.1E-16	3.9E-15	-0.50	70778918
Alpha-2-macroglobulin	A2M	500	-58.5 (-66.4, -48.7)	3.1E-16	1.0E-14	-0.50	66932947
Afamin	AFM	500	-38 (-44.7, -30.4)	4.1E-16	1.3E-14	-0.50	4501987
Attractin isoform 2	ATRN	500	-49.2 (-56.9, -40.2)	4.3E-16	1.3E-14	-0.50	21450863
Fibroblast activation protein, alpha subunit	FAP	423	-31.7 (-38.1, -24.7)	1.5E-14	4.6E-13	-0.51	16933540
Interferon-related developmental regulator 2	IFRD2	486	-29.9 (-36.1, -23.2)	2.2E-14	6.3E-13	-0.49	197333755
Apolipoprotein A-II	APOA2	500	-36.3 (-43.4, -28.5)	2.7E-14	7.7E-13	-0.49	4502149
Alpha 3 type vi collagen isoform 5	COL6A3	472	-38.9 (-46.2, -30.5)	4.8E-14	1.3E-12	-0.49	55743106
Alpha-2-hs-glycoprotein	AHSG	500	-39.3 (-46.9, -30.6)	2.4E-13	6.3E-12	-0.48	156523970
Serpin peptidase inhibitor, clade c, member 1	SERPINC1	500	-61.6 (-70.6, -49.9)	1.5E-12	3.5E-11	-0.47	4502261
Kelch-like protein 34	KLHL34	403	-36.7 (-44.2, -28.1)	1.7E-12	4.0E-11	-0.49	23397572
Peptidase inhibitor 16	PI16	500	-31.0 (-37.8, -23.4)	2.6E-12	5.7E-11	-0.47	70780384
Thrombospondin 4	THBS4	451	-25.5 (-31.4, -19.1)	2.7E-12	5.8E-11	-0.47	31543806
Gelsolin isoform a	GSN	493	-36.9 (-44.6, -28.2)	3.0E-12	6.3E-11	-0.48	4504165
Anthrax toxin receptor 2 isoform 1	ANTXR2	367	-33.4 (-40.6, -25.3)	3.3E-12	6.9E-11	-0.53	50513243
Retinol-binding protein 4, plasma	RBP4	500	-31.6 (-38.5, -23.8)	3.7E-12	7.4E-11	-0.47	55743122
Cd109 antigen isoform 2	CD109	300	-34.4 (-41.8, -26.1)	3.9E-12	7.8E-11	-0.56	227430301
Timp metallopeptidase inhibitor 2	TIMP2	368	-33.5 (-40.8, -25.3)	4.7E-12	9.2E-11	-0.49	4507511
Sparc-like protein 1	SPARCL1	493	-39.5 (-47.9, -29.7)	4.0E-11	7.5E-10	-0.46	190341024
Cadherin 5, type 2	CDH5	500	-34.1 (-41.9, -25.1)	1.1E-10	2.0E-09	-0.46	166362713
Olfactomedin related er localized protein isoform 1	OLFM1	465	-36.8 (-45.1, -27.2)	1.5E-10	2.6E-09	-0.47	17136143
Apolipoprotein M	APOM	500	-33.8 (-41.7, -24.8)	1.9E-10	3.4E-09	-0.46	22091452
Anthrax toxin receptor 1 isoform 1	ANTXR1	339	-28.1 (-35.1, -20.4)	2.4E-10	4.1E-09	-0.49	14149904
Plasma serine protease inhibitor	SERPINA5	500	-31.9 (-39.7, -23.1)	5.2E-10	8.8E-09	-0.45	194018472
Cadherin 13	CDH13	465	-34.4 (-42.6, -24.9)	7.6E-10	1.3E-08	-0.46	4502719
Tenascin xb isoform 1	TNXB	500	-29.5 (-37.1, -21.1)	1.2E-09	1.9E-08	-0.45	188528648
Dipeptidyl peptidase 4	DPP4	416	-35.3 (-43.9, -25.4)	2.0E-09	3.1E-08	-0.43	18765694
Cd93 antigen	CD93	416	-28.2 (-35.8, -19.7)	4.9E-09	7.4E-08	-0.47	88758613
Xaa-pro dipeptidase isoform 1	PEPD	466	-31.2 (-39.4, -21.9)	6.5E-09	9.7E-08	-0.45	149589008
Microtubule-actin cross-linking factor 1, isoforms 1/2/3/5 isoform a	MACF1	430	-28.2 (-35.8, -19.6)	6.6E-09	9.7E-08	-0.47	33188445
Procollagen c-endopeptidase enhancer 1	PCOLCE	500	-35.3 (-44.2, -25.0)	6.7E-09	9.8E-08	-0.44	157653329
Prenylcysteine oxidase 1	PCYOX1	493	-27.1 (-34.5, -18.8)	7.7E-09	1.1E-07	-0.44	166795301
Sulfhydryl oxidase 1 isoform a	QSOX1	500	-49.3 (-59.9, -36.0)	1.0E-08	1.5E-07	-0.44	13325075
Apolipoprotein H	APOH	500	-29.6 (-37.7, -20.5)	1.5E-08	2.1E-07	-0.44	153266841
Integrin alpha 2	ITGA2	187	-33.7 (-42.5, -23.5)	1.6E-08	2.1E-07	-0.49	116295258
Membrane alanine aminopeptidase	ANPEP	500	-35.2 (-44.5, -24.3)	4.0E-08	5.3E-07	-0.44	157266300
Inter-alpha (globulin) inhibitor h1 isoform a	ITIH1	500	-41.2 (-51.3, -28.9)	4.0E-08	5.3E-07	-0.44	156119625
Biotinidase	BTD	500	-35.5 (-44.9, -24.5)	4.2E-08	5.4E-07	-0.44	4557373
Insulin-like growth factor binding protein, acid labile subunit isoform 2	IGFALS	500	-23.7 (-30.7, -15.9)	4.3E-08	5.4E-07	-0.44	4826772
Phosphatidylinositol-glycan-specific phospholipase d isoform 1	GPLD1	500	-30.1 (-38.6, -20.5)	5.0E-08	6.3E-07	-0.44	29171717
Basement membrane-specific heparan sulfate proteoglycan core protein	HSPG2	493	-40.2 (-50.3, -28.0)	5.4E-08	6.7E-07	-0.43	126012571
Lymphatic vessel endothelial hyaluronic acid receptor 1	LYVE1	479	-22.9 (-29.8, -15.2)	6.8E-08	8.3E-07	-0.45	40549451
Butyrylcholinesterase	BCHE	500	-31.4 (-40.3, -21.2)	8.9E-08	1.1E-06	-0.44	4557351
Osteomodulin	OMD	465	-24.9 (-32.4, -16.5)	9.0E-08	1.1E-06	-0.47	4826876
Plasma kallikrein b1	KLKB1	500	-36.6 (-46.4, -25.0)	9.4E-08	1.1E-06	-0.44	78191798
Sex hormone-binding globulin isoform 1	SHBG	486	-19.6 (-25.9, -12.6)	2.3E-07	2.6E-06	-0.43	7382460
Multimerin-2	MMRN2	444	-26.3 (-34.5, -17.2)	2.7E-07	3.1E-06	-0.45	221316695
Mannan-binding lectin serine protease 1 isoform 2	MASP1	486	-26.6 (-34.8, -17.4)	2.9E-07	3.3E-06	-0.43	21264359
Paraoxonase 1	PON1	500	-21.1 (-28.0, -13.5)	3.7E-07	4.1E-06	-0.43	19923106
Neural cell adhesion molecule 1 isoform 3	NCAM1	453	-31.9 (-41.4, -20.8)	5.4E-07	5.8E-06	-0.45	115529478
Collagen, type vi, alpha 1	COL6A1	472	-24.3 (-32.2, -15.4)	7.9E-07	8.5E-06	-0.45	87196339

^a^Plasma proteins that achieved a Bonferroni corrected significance level (*P* <0.001/982 = 1.02e-06).

^b^The number of child plasma samples of each listed protein (50<n≤500).

^c^Percent change (%) in plasma AGP per two-fold (100%) increase in relative abundance of protein.

^d^ *P* value for the hypothesis test of null association between plasma AGP and protein.

^e^Adjusted *P* value correcting multiple hypothesis testing (false discovery rate).

^f^Correlation between plasma AGP concentration and protein.

^g^GenInfo Identifier for protein sequence assigned by the National Center for Biotechnology Information.

Separate verification analyses identified 41 (S3 Table) and 40 (S4 Table) proteins that were positively and negatively associated with CRP, respectively, passing the same Bonferroni corrected significance level as applied to the AGP analysis, of which 72 proteins were associated with both AGP and CRP. The 9 “non-overlapping” CRP-associated proteins (marked in red in S3 and S4 Tables) were still associated with AGP but less significantly (all *P* < 0.003). In addition, 27 proteins were differentially abundant between children with at least one episode of any morbidity symptom (fever, diarrhea, productive cough or rapid breathing) and children without any symptoms, passing a false discovery significance threshold of q <0.01 (S5 Table). All but one of the 27 proteins associated with morbidity symptoms were strongly correlated with AGP.

**Fig 2:**
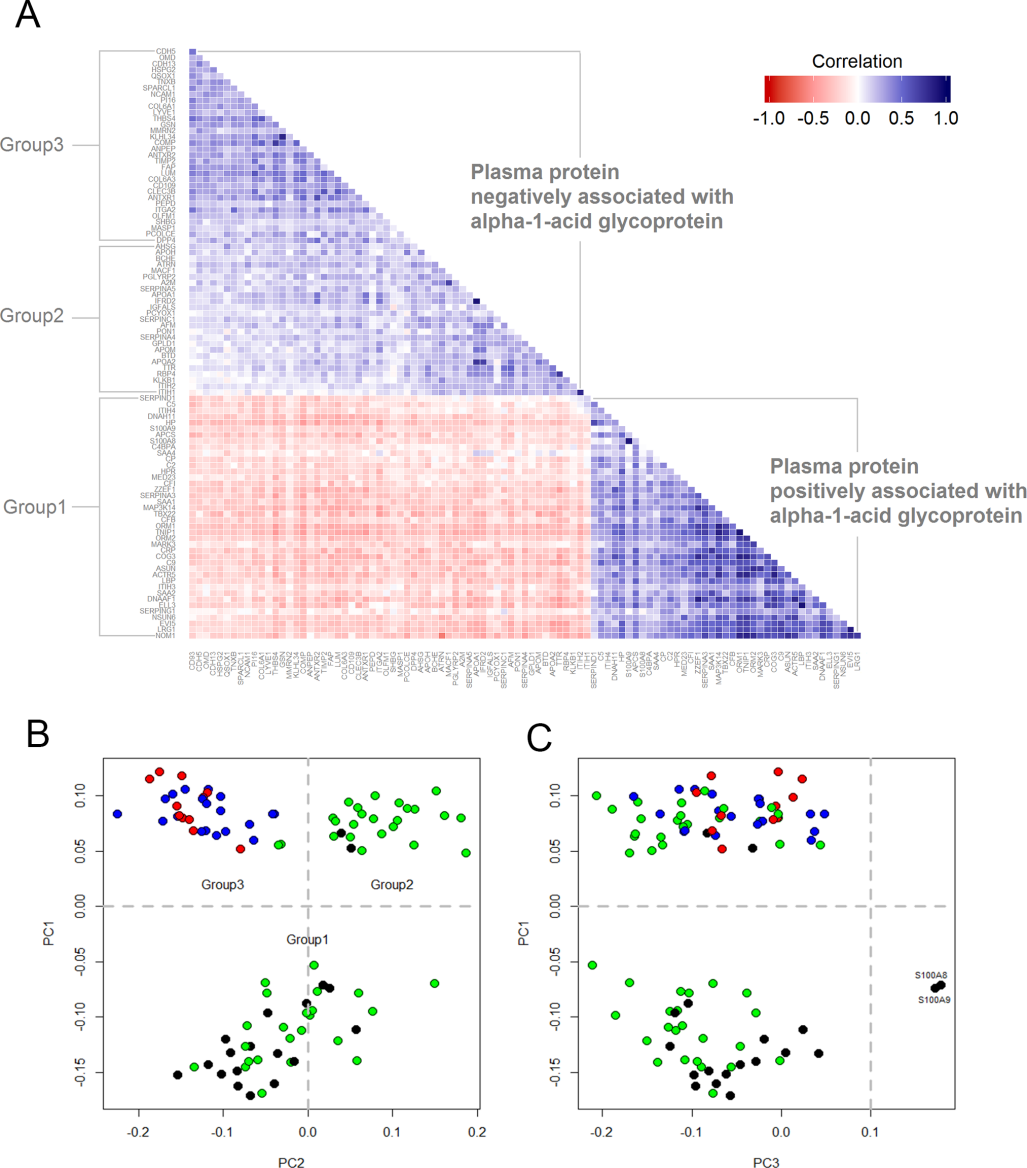
Correlation matrix and bi-plots from principal components (PC) analysis using plasma proteins associated with α-1-acid glycoprotein in 6–8 year old children in rural Nepal. (A) Bottom-triangle is the correlation matrix of plasma proteins positively associated with AGP (Group1). Upper-triangle is the correlation matrix of plasma protein negatively associated with AGP (Group 2 & 3). (B and C) Bi-plot was constructed by the first three principle components. Color depicts representative tissue origins or subcellular localization of proteins: black-intracellular space; green-hepatic origin and secreted into plasma; red-extracellular matrix; blue-extracellular matrix membrane binding. Proteins with PC1 less than 0 were assigned into group 1, proteins with PC1 and PC2 greater than 0 were assigned into group 2, and proteins with PC1 greater than 0 and PC2 less than 0 were assigned into group 3 (4 proteins were not included due to missing values and lack of information about subcellular localization).

We anticipated that proteins positively and negatively associated with AGP would also be correlated with each other. The correlation matrix, shown in Fig 2A, shows that plasma proteins positively associated with AGP were more highly correlated with each other (i.e., darker blue cells) than plasma proteins negatively associated with AGP (i.e., lighter blue cells). There were 83 and 5 protein-pairs, respectively, whose correlation coefficients (r) were greater than 0.6 within the positive and negative population plasma inflammasomes. Within the former, high correlations were observed among pairs involving ORM1-TNIP1-MAP3K14-COG3-ACTR5-NOM1 (all r >0.80), especially between LBP-ELL3 (r = 0.92), and LRG1-EVI5 (r = 0.86).

As expected, principal components (PC) analysis divided the population plasma inflammasome into proteins that were positively (PC1<0, referred to as Group 1) and negatively associated with AGP (PC1>0). (Fig 2B). However, two subgroups further emerged among proteins negatively associated with AGP. PC2 partitioned negatively associated proteins into two groups (Group 2 vs. Group 3) and PC3 separated two proteins (S100A8 and S100A9) from the rest of the proteins (Fig 2C). To better understand the unexpected partitioning among plasma proteins negatively correlated with AGP, we investigated cellular localization of the proteins. Proteins in PC2 are known to be mainly produced by the liver and secreted into the bloodstream (green) and proteins in PC3 are largely produced by extra-hepatic tissues and localized in extracellular matrix regions (red and blue).

### Localization and functions of plasma proteins associated AGP

Proteins associated with AGP are summarized by their most often described cellular localization and biological or molecular functions in Table 4. More than half of the proteins positively associated with AGP were primarily extracellular, secreted into circulation from the liver, and known to promote or regulate innate immune responses and inhibit oxidative activity. These included ORM1/2, CRP, SAA 1/2/4, bacterial lipopolysaccharide binding protein (LBP), LRG1 (a protein involved in granulocyte differentiation), components of the complement cascade, free hemoglobin scavengers, a copper-carrier, and several protease inhibitors. Other positively associated proteins are mainly localized in the membrane or intracellular space and involved in diverse functions, including leukocyte recruitment and trafficking, cell signaling, transcription, translation, DNA repair, protein methylation, modulation of cell cycle, cytokinesis and cytoskeleton, and endoplasmic reticulum-Golgi vesicle transport.

**Table 4:** Cellular localizati4on and molecular/biological functions of plasma proteins associated with α-1-acid glycoprotein (AGP) in 6–8 year old children in rural Nepal.^a^

Association	Cellular localization	Molecular/biological function	Protein
Positively associated with AGP (n = 40)	Extracellular (plasma) (n = 23)	Immune system	ORM1/2, APCS, LBP, LRG1
		Lipoproteins	SAA1/2/4
		Complement system	C2/5/9, CFB, CFI, C4BPA, CRP
		Transport/scavenger protein	HP/HPR, CP
		Serine proteases inhibitor	SERPINA3, SERPING1, SERPIND1, ITIH3/4
	Extracellular (plasma membrane) (n = 1)	Leukocyte-endothelial interaction	ITGAL
	Extra- & intra-cellular (n = 2)	Leukocyte trafficking	S100A8/9
	Intracellular (n = 14)	Regulation of cell signaling	MAP3K14, TNIP1
		Transcription and translation regulation, DNA/RNA binding	ELL3, NOM1, TBX22, MED23, NSUN6, ACTR5, MARK3
		Cell cycle, Cell division, Mitosis	EVI5, ASUN
		Cytoskeleton	DNAAF1, DNAH11
		ER-Golgi vesicle-mediated transport	COG3
Negatively associated with AGP (n = 56)	Extracellular (plasma) (n = 25)	Transport	RBP4, TTR, AFM, AHSG
		Lipoproteins	APOA1/2, APOH, APOM
		Serine proteinase inhibitor	SERPINA5, SERPINC1, A2M, SERPINA4, ITIH1/ ITIH2
		Serine type endopeptidase	KLKB1, MASP1
		Other enzymes	BCHE, PON1, BTD, PCYOX1, GPLD1
		Inflammatory response	ATRN
		Growth factor/Hormone binding	IGFALS, SHBG
		Peptidoglycan recognition	PGLYRP2
	Extracellular matrix (n = 11)	Collagen	COL6A1/3
		Non-collagenous glycoprotein	COMP, THBS4, TNXB, SPARCL1, MMRN2
		Proteoglycan	HSPG2, LUM
		Bone matrix, mineralization	CLEC3B, OMD
	Extracellular (plasma membrane) (n = 20)	Aminoprotease or peptidase	PCOLCE, PEPD, DPP4, FAP, ANPEP
		Cell-cell/cell-ECM adhesion	CDH13, CDH5, NCAM1, OLFM1, CD93, ANTRX1, ANTRX2, QSOX1, ITGA2
		Cytoskeleton modulation	GSN, MACF1
		Peptidase inhibitor	TIMP2, PI16, CD109
		Hyaluronan receptor	LYVE1

^a^Proteins of unknown function were not presented (KLHL34, IFRD2, and ZZEF1).

About half of the proteins negatively correlated with AGP (i.e., that decline in relative abundance with inflammation) are also considered hepatic proteins released into the bloodstream, but more involved in transport and metabolism of nutrients and small molecules (e.g., RBP4 and TTR for vitamin A; apolipoproteins A1/A2/H/M for lipid or cholesterol transport and metabolism; and AHSG for calcium and phosphate metabolism), sex hormone and growth factor binding (e.g., SHBG and IGFALS) or serine endopeptidase or proteinase inhibition in regulating blood coagulation and complement cascades, among other roles. Other negative correlates are physical constituents of the extracellular matrix (ECM), including collagens [e.g, collagen types VI α1 and 3 (COL6A1, COL6A3)], glycoproteins, glucosaminoglycans and bone matrix proteins. Proteins known to facilitate interaction between cells and ECM are aminoproteinases/peptidases (e.g. PCOLCE and PEPD), protease inhibitors (e.g. TIMP2) and numerous cell-cell or cell-matrix adhesion molecules (e.g. CDH5 and ANTRX1).

## Discussion

This study provides evidence of a population plasma inflammasome, defined in relation to the continuous distribution of an established biomarker of inflammation, α-1-acid glycoprotein. We virtually ensured identification of nearly 100 plasma proteins associated with AGP by applying a stringent family-wise error rate threshold of 0.1%. Approximately three-quarters of the same proteins were similarly correlated with a second acute phase reactant in plasma, CRP. Study children in rural Nepal were undernourished, similar to many child populations in rural Asia, but were active and not acutely ill, with only 14% reporting any illness symptom in the previous week. As such, the set of proteins observed to covary with AGP can be inferred to reflect homeostatic control of inflammation within the environment of this typical South Asian rural setting.

Positive plasma inflammasome proteins exhibited stronger associations with AGP and greater variation in their degree of change per unit difference in AGP concentration than negatively associated proteins, possibly reflecting higher metabolic priority and functional specificity. In addition to established acute phase proteins, our quantitative proteomics approach identified numerous intracellular signaling, membrane-bound, and extracellular matrix molecules not widely regarded as acute phase reactants, appearing to reflect a systemic repertoire of proteins that respond to inflammation.

Acute phase proteins, complement components, protease inhibitors and transport proteins with anti-oxidant activity positively covaried with plasma AGP, in accordance with their expected roles in responding to stress [1]. Many of these biomarkers are produced in the liver and secreted into plasma [10]. As AGP abundance in plasma is attributable to expression of homologous *ORM1 (AGP1)* and *ORM2 (AGP2)* genes [42], ORM1 and ORM2 were expected, and observed, to be among the strongest correlates of AGP (measured by radial immunodiffusion), offering evidence that mass spectrometry is a valid method to detect and quantify relative protein abundance. LRG1, HP, SERPINA3, CRP, SAA1, C9, and LBP were positively associated with AGP (all *P* <1.0x10^-25^), suggesting these proteins increase in circulation during inflammation [10]. Substantial variation in the strength of association with AGP likely reflects wide differences in expression across acute and chronic phase proteins [43–45]. It is likely that rises in complement components, LRG1 and SERPINA3 reflect a persistent inflammation that accompanies a modest elevation in AGP where exposure to parasites, bacteria [46–48] and environmental toxins, such as aflatoxin and arsenic, are common [49, 50]. Positively associated proteins we observed are known to largely be involved in immune activation (e.g., CRP, SAA, LBP, and complement components), proteolytic attack processes (SERPINA3, SERPING1, and SEPRIND1), and transport of pro-oxidative metabolites (CP and HP) [44]. ORM1/2 and LRG1 are involved in immunomodulation and granulocyte differentiation, respectively, although their molecular roles have not been fully elucidated [51–53]. Collectively, our results suggest that hepatic-driven proteins that positively covary with inflammation are involved in host defense.

Fourteen of 41 positive correlates of AGP are intracellular proteins whose larger numbers of missing values (Table 2) support the notion that these proteins may be low in abundance, and leaked or secreted from tissues as part of normal metabolism and tissue maintenance. The strongest positive correlate (*P* = 7.6x10^-112^), sharing a nearly 1:1 association with AGP, was TNIP1, suggesting their co-regulation during inflammation, although no study to our knowledge has drawn this direct metabolic linkage. TNIP1 regulates inflammation by inhibiting cell signal transduction such as in the NF-kappa-B activation pathway [54]. However, unknown extracellular functions of TNIP1 leave its positive association with AGP in plasma unexplained. A 30~60% increase in AGP concentration was associated with a 100% increase in intracellular proteins involved in signal transduction, and protein transcription, translation, maturation and secretion. High correlations between intracellular proteins and circulating ORM1, LBP, and LRG1 suggest their abundance in plasma could reflect biosynthesis of inflammatory mediators [55], or act themselves as acute phase proteins.

Among proteins negatively correlated with plasma AGP, about half are known to transport and regulate bioavailability of nutrients and hormones. Correlations among these proteins were negligible, suggesting independent regulation and metabolic pathways, while still being susceptible to hepatic-directed reduction during inflammation [56]. Some negative correlates are components of lipoprotein particles, involved in anti-inflammation (APOA1/2), antioxidant functions (PON1, PON3, and PCYOX1) and reverse cholesterol transport (PLTP, CLU and LCAT) (all *P* <1.0x10^-4^). These observations coupled with those of increased abundance in pro-inflammatory serum amyloid A (SAAs) apoliporoteins with AGP support considerable alterations in plasma lipoprotein composition during homeostatic regulation of inflammation [57]. RBP4, TTR, AHSG, and APOA1/2 are well-known negative acute phase proteins [10] that are consistent with known redistributions of vitamin A, calcium, phosphate and lipids during inflammation [57–60].

In our analysis, a small decrement in AGP per two-fold increment in relative protein abundance identifies proteins that are likely to decrease markedly during acute inflammation. The smallest decrease in AGP (20–24%) was observed with a two-fold rise in sex hormone binding globulin (SHBG), insulin-like growth factor (IGF) acid labile subunit and IGF binding protein (BP) 3 (all *P* <1.0x10^-5^), consistent with expected reductions in insulin-like growth factor 1 and androgens during inflammation [10, 61]. IGFALS and IGFBP3 form a ternary complex with most plasma IGFs which regulate somatic growth and development [62, 63]. SHBG binds and regulates circulating androgens and estrogens [64]. Population studies have revealed inverse associations between SHBG and inflammatory markers and adiposity-related early onset of puberty among girls [65, 66]. Observed inverse associations between AGP and hepatic proteins may reflect metabolic adaptation to altered endocrine signaling in response to inflammation.

Proteins that serve as components or regulators of the extracellular matrix (ECM) were negatively associated with inflammation [67]. Our PCA results revealed that variance in the abundance of these proteins differed from those of hepatic origin, possibly due to differences in intravascular concentration between classic plasma and extravascular proteins. Lumican (LUM), cartilage oligomeric matrix protein (COMP), tetranectin (CLEC3B), osteomodulin (OMD), and collagen α-1 and -3 type VI (COL6A1/3) are enriched in cartilage, bone matrix, skeletal muscle and adipose tissues [68–72]. Beyond structural and functional components of ECM, many cell surface molecules or enzymatic proteins are involved in penetrating the vascular endothelial cells, regulation of pericellular proteolysis of ECM, and cell migration into inflamed tissues, critical to tissue repair and turnover [73–81]. Our results suggest that proteins that maintain integrity of the ECM are down-regulated, possibly reflecting metabolic rebalancing between host defense and healing mechanisms [82]. These proteins are particularly important in chronic inflammatory conditions that commonly accompany degradation of connective tissue [83–86].

Our findings corroborate those in -omics studies in animals that have examined changes in gene or protein expression levels of acute phase proteins during inflammation. Yoo *et al*. showed in mice that 898 out of 8,551 protein-encoding genes (~7%) in the hepatic transcriptome were altered, equally up and down, by endotoxin-induced inflammation [87]. Similarly, we observed that half of the population plasma inflammasome was primarily hepatic in origin, equally divided across positive and negative correlates of AGP, and possibly reflecting a need to maintain protein equilibria in the vascular compartment [44]. Kelly-Spratt *et al*. reported that a third of ~500 plasma proteins detected in mice increased or decreased by more than 1.25 fold in response to induced-inflammation [22]. We observed that a large fraction (~20%) of our measured plasma proteome covaried with inflammation, at a false discovery rate below 1% (S1 and S2 Tables). The study also showed that induced-inflammation reduced the abundance of proteins involved in ECM and collagen network remodeling [22], which were also similarly observed in the present study.

The diversity of plasma proteins observed to be associated with AGP in this ambient population offer an unbiased view of inflammation. With this large-scale and untargeted approach, we identified potential networks of interaction and a large number of candidate biomarkers. Defining the plasma inflammasome with respect to AGP, an established index of subclinical inflammation, enabled us to identify proteins that are likely relevant to the homeostatic response to inflammation. Results of additional analyses in relation to plasma CRP concentration and children’s recent morbidity offer further support of the validity of the population plasma inflammasome indexed by AGP. While our unit of estimated protein amount, i.e., relative abundance, restricts ability to directly apply these findings, biomarkers of strongest association can be considered candidates for absolute quantification to expand the repertoire of biomarkers that reflect diverse mechanisms and possibly sources inflammation. However, with a cross-sectional design, we could not infer metabolic proximity or causality of association among proteins. We also did not have information about specific parasitic or bacterial infection or environmental toxins that could reveal greater specificity of protein associations with causal agents. We depleted, but did not completely remove, plasma samples of 6 highly abundant proteins, and the employed iTRAQ technology could not quantify low abundant cytokines and chemokines which mediate inflammation. These limitations reveal challenges of profiling a whole plasma inflammasome of a likely vast dynamic range in protein abundance. However, it may be promising to investigate and integrate less abundant proteins from different spectrums of abundance to build a more complete profile of a population plasma inflammasome. Lastly, although many proteins are involved in non-specific response to inflammation, the findings of this study will be most likely generalizable to populations living in areas where undernutrition, infections and environmental hazards are common.

## Conclusions

This study provides evidence of strong association between an index biomarker of chronic inflammation and proteins of host defense, nutrient and hormonal metabolism and tissue remodeling. It is tempting to speculate that the low-grade inflammation seen in this study of young children could reflect mild pathological processes early in life, and thus risk, of adult chronic diseases of rising prominence in impoverished societies of South Asia.

## Supporting Information

S1 Table Plasma Proteins Positively Associated with α-1-Acid Glycoprotein (AGP) in 500 children (q<0.01, proteins ordered by q). (XLSX)

S2 Table Plasma Proteins Negatively Associated with α-1-Acid Glycoprotein (AGP) in 500 children (q<0.01, proteins ordered by q). (XLSX)

S3 Table Plasma Proteins Positively Associated with C-reactive protein (CRP) in 500 children. (XLSX)

S4 Table Plasma Proteins Negatively Associated with C-reactive protein (CRP) in 500 children. (XLSX)

S5 Table Differentially abundant plasma proteins between children with at least one morbidity episode and children without any symptoms (q<0.01, proteins ordered by q). (XLSX)

## References

[1] Kushner I. The phenomenon of the acute phase response. Ann N Y Acad Sci. 1982;389:39–48. 7046585 10.1111/j.1749-6632.1982.tb22124.x

[2] Ceciliani F, Giordano A, Spagnolo V. The systemic reaction during inflammation: the acute-phase proteins. Protein Pept Lett. 2002;9(3):211–223. 12144517 10.2174/0929866023408779

[3] Kumar V, Abbas AK, Aster JC. Inflammation and Repair In: Kumar V, Abbas AK, Aster JC, editors. Robbins Basic Pathology. 9th ed Philadelphia, U.S.: ELSEVIER Saunders; 2012.

[4] Nathan C, Ding A. Nonresolving inflammation. Cell. 2010;140(6):871–882. 10.1016/j.cell.2010.02.029 20303877

[5] Mal-Ed Network Investigators. The MAL-ED study: a multinational and multidisciplinary approach to understand the relationship between enteric pathogens, malnutrition, gut physiology, physical growth, cognitive development, and immune responses in infants and children up to 2 years of age in resource-poor environments. Clin Infect Dis. 2014;59 Suppl 4:S193–206. 10.1093/cid/ciu653 25305287

[6] Prendergast AJ, Rukobo S, Chasekwa B, Mutasa K, Ntozini R, Mbuya MN, et al Stunting is characterized by chronic inflammation in Zimbabwean infants. PLoS One. 2014;9(2):e86928 10.1371/journal.pone.0086928 24558364 PMC3928146

[7] Rodriguez L, Cervantes E, Ortiz R. Malnutrition and gastrointestinal and respiratory infections in children: a public health problem. Int J Environ Res Public Health. 2011;8(4):1174–1205. 10.3390/ijerph8041174 21695035 PMC3118884

[8] Ahmed S, Moore SE, Kippler M, Gardner R, Hawlader MD, Wagatsuma Y, et al Arsenic exposure and cell-mediated immunity in pre-school children in rural bangladesh. Toxicol Sci. 2014;141(1):166–175. 10.1093/toxsci/kfu113 24924402 PMC4833103

[9] Turner PC. The molecular epidemiology of chronic aflatoxin driven impaired child growth. Scientifica (Cairo). 2013;2013:152879. 24455429 10.1155/2013/152879 PMC3881689

[10] Gabay C, Kushner I. Acute-phase proteins and other systemic responses to inflammation. N Engl J Med. 1999;340(6):448–454. 9971870 10.1056/NEJM199902113400607

[11] AmiGO2. acute-phase response (GO:0006953): the Gene Ontology; 2014. 2.1.4. Available: http://amigo.geneontology.org/amigo/term/GO:0006953/.

[12] Whicher T, Bienvenu J, Price CP. Molecular biology, measurement and clinical utility of the acute phase proteins. Pure and Applied Chemistry. 1991;63(8):1111–1116.

[13] Schulze KJ, Christian P, Wu LS, Arguello M, Cui H, Nanayakkara-Bind A, et al Micronutrient deficiencies are common in 6- to 8-year-old children of rural Nepal, with prevalence estimates modestly affected by inflammation. J Nutr. 2014;144(6):979–987. 10.3945/jn.114.192336 24744314 PMC4018957

[14] Thurnham DI, McCabe GP. Influence of infection and inflammation on biomarkers of nutritional status with an emphasis on vitamin A and iron In: World Health Organization, editor. Priorities in the Assessment of Vitamin A and Iron Status in Populations. Geneva, Switzerland: World Health Organization; 2012 p. 63–80.

[15] Raiten DJ, Sakr Ashour FA, Ross AC, Meydani SN, Dawson HD, Stephensen CB, et al Inflammation and Nutritional Science for Programs/Policies and Interpretation of Research Evidence (INSPIRE). J Nutr. 2015;145(5):1039S–1108S. 10.3945/jn.114.194571 25833893 PMC4448820

[16] Ceciliani F, Pocacqua V. The acute phase protein alpha1-acid glycoprotein: a model for altered glycosylation during diseases. Curr Protein Pept Sci. 2007;8(1):91–108. 17305563 10.2174/138920307779941497

[17] Cole RN, Ruczinski I, Schulze K, Christian P, Herbrich S, Wu L, et al The plasma proteome identifies expected and novel proteins correlated with micronutrient status in undernourished Nepalese children. J Nutr. 2013;143(10):1540–1548. 10.3945/jn.113.175018 23966331 PMC6879017

[18] Yuasa I, Umetsu K, Suenaga K, Robinet-Levy M. Orosomucoid (ORM) typing by isoelectric focusing: evidence of two structural loci ORM1 and ORM2. Hum Genet. 1986;74(2):160–161. 3770743 10.1007/BF00282080

[19] Anderson NL, Anderson NG. The human plasma proteome: history, character, and diagnostic prospects. Mol Cell Proteomics. 2002;1(11):845–867. 12488461 10.1074/mcp.r200007-mcp200

[20] Kalmovarin N, Friedrichs WE, O'Brien HV, Linehan LA, Bowman BH, Yang F. Extrahepatic expression of plasma protein genes during inflammation. Inflammation. 1991;15(5):369–379. 1757124 10.1007/BF00917353

[21] Petrilli V, Papin S, Tschopp J. The inflammasome. Curr Biol. 2005;15(15):R581 16085473 10.1016/j.cub.2005.07.049

[22] Kelly-Spratt KS, Pitteri SJ, Gurley KE, Liggitt D, Chin A, Kennedy J, et al Plasma proteome profiles associated with inflammation, angiogenesis, and cancer. PLoS One. 2011;6(5):e19721 10.1371/journal.pone.0019721 21589862 PMC3093388

[23] Saha S, Harrison SH, Chen JY. Dissecting the human plasma proteome and inflammatory response biomarkers. Proteomics. 2009;9(2):470–484. 10.1002/pmic.200800507 19105179 PMC3402908

[24] Qian WJ, Jacobs JM, Camp DG 2nd, Monroe ME, Moore RJ, Gritsenko MA, et al Comparative proteome analyses of human plasma following in vivo lipopolysaccharide administration using multidimensional separations coupled with tandem mass spectrometry. Proteomics. 2005;5(2):572–584. 15627965 10.1002/pmic.200400942 PMC1781926

[25] Calder PC, Ahluwalia N, Albers R, Bosco N, Bourdet-Sicard R, Haller D, et al A consideration of biomarkers to be used for evaluation of inflammation in human nutritional studies. Br J Nutr. 2013;109 Suppl 1:S1–34. 10.1017/S0007114512005119 23343744

[26] Christian P, Khatry SK, Katz J, Pradhan EK, LeClerq SC, Shrestha SR, et al Effects of alternative maternal micronutrient supplements on low birth weight in rural Nepal: double blind randomised community trial. BMJ. 2003;326(7389):571 12637400 10.1136/bmj.326.7389.571 PMC151518

[27] Stewart CP, Christian P, Schulze KJ, Leclerq SC, West KP Jr., Khatry SK. Antenatal micronutrient supplementation reduces metabolic syndrome in 6- to 8-year-old children in rural Nepal. J Nutr. 2009;139(8):1575–1581. 10.3945/jn.109.106666 19549749

[28] Stewart CP, Christian P, LeClerq SC, West KP Jr., Khatry SK. Antenatal supplementation with folic acid + iron + zinc improves linear growth and reduces peripheral adiposity in school-age children in rural Nepal. Am J Clin Nutr. 2009;90(1):132–140. 10.3945/ajcn.2008.27368 19474130 PMC2696997

[29] Herbrich SM, Cole RN, West KP Jr., Schulze K, Yager JD, Groopman JD, et al Statistical inference from multiple iTRAQ experiments without using common reference standards. J Proteome Res. 2013;12(2):594–604. 10.1021/pr300624g 23270375 PMC4223774

[30] Harville DA. Maximum Likelihood Approaches to Variance Component Estimation and to Related Problems. Journal of the American Statistical Association. 1977;72(358):320–338.

[31] Robinson GK. That BLUP is a good thing: the estimation of random effects. Stat Sci. 1991;6:15–32.

[32] Luo R, Zhao H. Protein quantitation using iTRAQ: Review on the sources of variations and analysis of nonrandom missingness. Stat Interface. 2012;5(1):99–107. 23888187 10.4310/sii.2012.v5.n1.a9 PMC3719432

[33] Gower JC, Lubbe SG, Roux NJL. Understanding Biplots: Wiley; 2011.

[34] Gray KA, Yates B, Seal RL, Wright MW, Bruford EA. Genenames.org: the HGNC resources in 2015. Nucleic Acids Res. 2015;43(Database issue):D1079–1085. 10.1093/nar/gku1071 25361968 PMC4383909

[35] Binder JX, Pletscher-Frankild S, Tsafou K, Stolte C, O'Donoghue SI, Schneider R, et al COMPARTMENTS: unification and visualization of protein subcellular localization evidence. Database (Oxford). 2014;2014:bau012. 24573882 10.1093/database/bau012 PMC3935310

[36] Geer LY, Marchler-Bauer A, Geer RC, Han L, He J, He S, et al The NCBI BioSystems database. Nucleic Acids Res. 2010;38(Database issue):D492–496. 10.1093/nar/gkp858 19854944 PMC2808896

[37] Huntley RP, Sawford T, Mutowo-Meullenet P, Shypitsyna A, Bonilla C, Martin MJ, et al The GOA database: Gene Ontology annotation updates for 2015. Nucleic Acids Res. 2015;43(Database issue):D1057–1063. 10.1093/nar/gku1113 25378336 PMC4383930

[38] UniProt C. Activities at the Universal Protein Resource (UniProt). Nucleic Acids Res. 2014;42(Database issue):D191–198. 10.1093/nar/gkt1140 24253303 PMC3965022

[39] Wu C, Orozco C, Boyer J, Leglise M, Goodale J, Batalov S, et al BioGPS: an extensible and customizable portal for querying and organizing gene annotation resources. Genome Biol. 2009;10(11):R130 10.1186/gb-2009-10-11-r130 19919682 PMC3091323

[40] de Onis M, Onyango AW, Borghi E, Siyam A, Nishida C, Siekmann J. Development of a WHO growth reference for school-aged children and adolescents. Bull World Health Organ. 2007;85(9):660–667. 18026621 10.2471/BLT.07.043497 PMC2636412

[41] Thurnham DI, Mburu AS, Mwaniki DL, De Wagt A. Micronutrients in childhood and the influence of subclinical inflammation. Proc Nutr Soc. 2005;64(4):502–509. 16313694 10.1079/pns2005468

[42] Nakamura H, Yuasa I, Umetsu K, Nakagawa M, Nanba E, Kimura K. The rearrangement of the human alpha(1)-acid glycoprotein/orosomucoid gene: evidence for tandemly triplicated genes consisting of two AGP1 and one AGP2. Biochem Biophys Res Commun. 2000;276(2):779–784. 11027547 10.1006/bbrc.2000.3522

[43] Kushner I, Mackiewicz A. Acute phase proteins as disease markers. Dis Markers. 1987;5(1):1–11. 2458880

[44] Mackiewicz A, Kushner I, Baumann H. Acute Phase Proteins: Molecular Biology, Biochemistry, and Clinical Applications. Florida: CRC Press; 1993.

[45] Samols D, Agrawal A, Kushner I. Acute Phase Proteins. Cytokine references online [Internet]. 2002. Available: https://www.researchgate.net/profile/Irving_Kushner/publication/228050478_Acutephase_Proteins/links/00b7d51597e03b5192000000.pdf.

[46] Navitsky RC, Dreyfuss ML, Shrestha J, Khatry SK, Stoltzfus RJ, Albonico M. Ancylostoma duodenale is responsible for hookworm infections among pregnant women in the rural plains of Nepal. J Parasitol. 1998;84(3):647–651. 9645880

[47] Chowdhury R, Huda MM, Kumar V, Das P, Joshi AB, Banjara MR, et al The Indian and Nepalese programmes of indoor residual spraying for the elimination of visceral leishmaniasis: performance and effectiveness. Ann Trop Med Parasitol. 2011;105(1):31–35. 10.1179/136485911X12899838683124 21294947 PMC4089790

[48] Coles CL, Sherchand JB, Khatry SK, Katz J, Leclerq SC, Mullany LC, et al Nasopharyngeal carriage of S. pneumoniae among young children in rural Nepal. Trop Med Int Health. 2009;14(9):1025–1033. 10.1111/j.1365-3156.2009.02331.x 19563428 PMC2770711

[49] Groopman JD, Egner PA, Schulze KJ, Wu LS, Merrill R, Mehra S, et al Aflatoxin exposure during the first 1000 days of life in rural South Asia assessed by aflatoxin B(1)-lysine albumin biomarkers. Food Chem Toxicol. 2014;74:184–189. 10.1016/j.fct.2014.09.016 25308602 PMC4322911

[50] Thakur JK, Thakur RK, Ramanathan A, Kumar M, Singh SK. Arsenic Contamination of Groundwater in Nepal—An Overview. Water. 2010;3(1):1–20.

[51] Hochepied T, Berger FG, Baumann H, Libert C. Alpha(1)-acid glycoprotein: an acute phase protein with inflammatory and immunomodulating properties. Cytokine Growth Factor Rev. 2003;14(1):25–34. 12485617 10.1016/s1359-6101(02)00054-0

[52] Wang X, Abraham S, McKenzie JA, Jeffs N, Swire M, Tripathi VB, et al LRG1 promotes angiogenesis by modulating endothelial TGF-beta signalling. Nature. 2013;499(7458):306–311. 10.1038/nature12345 23868260 PMC3836402

[53] O'Donnell LC, Druhan LJ, Avalos BR. Molecular characterization and expression analysis of leucine-rich alpha2-glycoprotein, a novel marker of granulocytic differentiation. J Leukoc Biol. 2002;72(3):478–485. 12223515

[54] Ramirez VP, Gurevich I, Aneskievich BJ. Emerging roles for TNIP1 in regulating post-receptor signaling. Cytokine Growth Factor Rev. 2012;23(3):109–118. 10.1016/j.cytogfr.2012.04.002 22542476 PMC3366012

[55] Fey GH, Gauldie J. The acute phase response of the liver in inflammation. Prog Liver Dis. 1990;9:89–116. 1690438

[56] Aldred AR, Schreiber G. The Negative Acute Phase Proteins In: Mackiewicz A, Kushner I, Baumann H, editors. Acute Phase Proteins Molecular Biology, Biochemistry, and Clinical Applications: Taylor & Francis; 1993 p. 21–38.

[57] Khovidhunkit W, Kim MS, Memon RA, Shigenaga JK, Moser AH, Feingold KR, et al Effects of infection and inflammation on lipid and lipoprotein metabolism: mechanisms and consequences to the host. J Lipid Res. 2004;45(7):1169–1196. 15102878 10.1194/jlr.R300019-JLR200

[58] Moody BJ. Changes in the serum concentrations of thyroxine-binding prealbumin and retinol-binding protein following burn injury. Clin Chim Acta. 1982;118(1):87–92. 6797761 10.1016/0009-8981(82)90229-7

[59] Rosales FJ, Ross AC. Acute inflammation induces hyporetinemia and modifies the plasma and tissue response to vitamin A supplementation in marginally vitamin A-deficient rats. J Nutr. 1998;128(6):960–966. 9614154 10.1093/jn/128.6.960

[60] Jahnen-Dechent W, Heiss A, Schafer C, Ketteler M. Fetuin-A regulation of calcified matrix metabolism. Circ Res. 2011;108(12):1494–1509. 10.1161/CIRCRESAHA.110.234260 21659653

[61] Tsilidis KK, Rohrmann S, McGlynn KA, Nyante SJ, Lopez DS, Bradwin G, et al Association between endogenous sex steroid hormones and inflammatory biomarkers in US men. Andrology. 2013;1(6):919–928. 10.1111/j.2047-2927.2013.00129.x 24124163 PMC3812341

[62] Le Roith D, Bondy C, Yakar S, Liu JL, Butler A. The somatomedin hypothesis: 2001. Endocr Rev. 2001;22(1):53–74. 11159816 10.1210/edrv.22.1.0419

[63] Baxter RC. Insulin-like growth factor binding proteins in the human circulation: a review. Horm Res. 1994;42(4–5):140–144. 7532612 10.1159/000184186

[64] Anderson DC. Sex-hormone-binding globulin. Clin Endocrinol (Oxf). 1974;3(1):69–96. 4134992 10.1111/j.1365-2265.1974.tb03298.x

[65] Liao CH, Li HY, Yu HJ, Chiang HS, Lin MS, Hua CH, et al Low serum sex hormone-binding globulin: marker of inflammation? Clin Chim Acta. 2012;413(7–8):803–807. 10.1016/j.cca.2012.01.021 22293276

[66] Pinkney J, Streeter A, Hosking J, Mohammod M, Jeffery A, Wilkin T. Adiposity, chronic inflammation, and the prepubertal decline of sex hormone binding globulin in children: evidence for associations with the timing of puberty (earlybird 58). J Clin Endocrinol Metab. 2014;99(9):3224–3232. 10.1210/jc.2013-3902 24926948

[67] Hynes RO. The extracellular matrix: not just pretty fibrils. Science. 2009;326(5957):1216–1219. 10.1126/science.1176009 19965464 PMC3536535

[68] Byron A, Humphries JD, Humphries MJ. Defining the extracellular matrix using proteomics. Int J Exp Pathol. 2013;94(2):75–92. 10.1111/iep.12011 23419153 PMC3607136

[69] Wilson R. The extracellular matrix: an underexplored but important proteome. Expert Rev Proteomics. 2010;7(6):803–806. 10.1586/epr.10.93 21142880

[70] Wewer UM, Ibaraki K, Schjorring P, Durkin ME, Young MF, Albrechtsen R. A potential role for tetranectin in mineralization during osteogenesis. J Cell Biol. 1994;127(6 Pt 1):1767–1775. 7798325 10.1083/jcb.127.6.1767 PMC2120295

[71] Khan T, Muise ES, Iyengar P, Wang ZV, Chandalia M, Abate N, et al Metabolic dysregulation and adipose tissue fibrosis: role of collagen VI. Mol Cell Biol. 2009;29(6):1575–1591. 10.1128/MCB.01300-08 19114551 PMC2648231

[72] Urciuolo A, Quarta M, Morbidoni V, Gattazzo F, Molon S, Grumati P, et al Collagen VI regulates satellite cell self-renewal and muscle regeneration. Nat Commun. 2013;4:1964 10.1038/ncomms2964 23743995 PMC3682802

[73] Arora PD, Glogauer M, Kapus A, Kwiatkowski DJ, McCulloch CA. Gelsolin mediates collagen phagocytosis through a rac-dependent step. Mol Biol Cell. 2004;15(2):588–599. 14617805 10.1091/mbc.E03-07-0468 PMC329256

[74] Bell SE, Mavila A, Salazar R, Bayless KJ, Kanagala S, Maxwell SA, et al Differential gene expression during capillary morphogenesis in 3D collagen matrices: regulated expression of genes involved in basement membrane matrix assembly, cell cycle progression, cellular differentiation and G-protein signaling. J Cell Sci. 2001;114(Pt 15):2755–2773. 11683410 10.1242/jcs.114.15.2755

[75] Fukasawa K, Fujii H, Saitoh Y, Koizumi K, Aozuka Y, Sekine K, et al Aminopeptidase N (APN/CD13) is selectively expressed in vascular endothelial cells and plays multiple roles in angiogenesis. Cancer Lett. 2006;243(1):135–143. 16466852 10.1016/j.canlet.2005.11.051

[76] Hotchkiss KA, Basile CM, Spring SC, Bonuccelli G, Lisanti MP, Terman BI. TEM8 expression stimulates endothelial cell adhesion and migration by regulating cell-matrix interactions on collagen. Exp Cell Res. 2005;305(1):133–144. 15777794 10.1016/j.yexcr.2004.12.025

[77] Kim SH, Turnbull J, Guimond S. Extracellular matrix and cell signalling: the dynamic cooperation of integrin, proteoglycan and growth factor receptor. J Endocrinol. 2011;209(2):139–151. 10.1530/JOE-10-0377 21307119

[78] Korpos E, Wu C, Sorokin L. Multiple roles of the extracellular matrix in inflammation. Curr Pharm Des. 2009;15(12):1349–1357. 19355973 10.2174/138161209787846685

[79] Man XY, Finnson KW, Baron M, Philip A. CD109, a TGF-beta co-receptor, attenuates extracellular matrix production in scleroderma skin fibroblasts. Arthritis Res Ther. 2012;14(3):R144 10.1186/ar3877 22694813 PMC3446527

[80] O'Brien P O'Connor BF. Seprase: an overview of an important matrix serine protease. Biochim Biophys Acta. 2008;1784(9):1130–1145. 10.1016/j.bbapap.2008.01.006 18262497

[81] Yucel G, Oro AE. Cell migration: GSK3beta steers the cytoskeleton's tip. Cell. 2011;144(3):319–321. 10.1016/j.cell.2011.01.023 21295692 PMC3929416

[82] Schreiber G. Synthesis and secretion of APP In: Glaumann H, Theodore P, Redman C, editors. Plasma protein secretion by the liver. London: Academic Press; 1983.

[83] Redlich K, Smolen JS. Inflammatory bone loss: pathogenesis and therapeutic intervention. Nat Rev Drug Discov. 2012;11(3):234–250. 10.1038/nrd3669 22378270

[84] Ademowo OS, Staunton L, FitzGerald O, Pennington SR. Biomarkers of inflammatory arthritis and proteomics In: Stanilova SA, editor. Genes and Autoimmunity—Intracellular Signaling and Microbiome Contribution: InTech; 2013 p. 237–267.

[85] Delano MJ, Moldawer LL. The origins of cachexia in acute and chronic inflammatory diseases. Nutr Clin Pract. 2006;21(1):68–81. 16439772 10.1177/011542650602100168

[86] Tseng S, Reddi AH, Di Cesare PE. Cartilage Oligomeric Matrix Protein (COMP): A Biomarker of Arthritis. Biomark Insights. 2009;4:33–44. 19652761 10.4137/bmi.s645 PMC2716683

[87] Yoo JY, Desiderio S. Innate and acquired immunity intersect in a global view of the acute-phase response. Proc Natl Acad Sci U S A. 2003;100(3):1157–1162. 12540827 10.1073/pnas.0336385100 PMC298743

